# Carbon stocks and changes of dead organic matter in China's forests

**DOI:** 10.1038/s41467-017-00207-1

**Published:** 2017-07-28

**Authors:** Jianxiao Zhu, Huifeng Hu, Shengli Tao, Xiulian Chi, Peng Li, Lai Jiang, Chengjun Ji, Jiangling Zhu, Zhiyao Tang, Yude Pan, Richard A. Birdsey, Xinhua He, Jingyun Fang

**Affiliations:** 1 Department of Ecology, College of Urban and Environmental Science, and Key Laboratory for Earth Surface Processes of the Ministry of Education, Peking University, Beijing, 100871 China; 2 State Key Laboratory of Vegetation and Environmental Change, Institute of Botany, Chinese Academy of Sciences, Beijing, 100093 China; 3 US Department of Agriculture Forest Service, Newtown Square, PA 19073 USA; 4 Woods Hole Research Center, Falmouth, MA, 02540 USA; 5 Centre of Excellence for Soil Biology, College of Resources and Environment, Southwest University, Chongqing, 400715 China

## Abstract

Forests play an important role in global carbon cycles. However, the lack of available information on carbon stocks in dead organic matter, including woody debris and litter, reduces the reliability of assessing the carbon cycles in entire forest ecosystems. Here we estimate that the national DOM carbon stock in the period of 2004–2008 is 925 ± 54 Tg, with an average density of 5.95 ± 0.35 Mg C ha^−1^. Over the past two decades from periods of 1984−1988 to 2004−2008, the national dead organic matter carbon stock has increased by 6.7 ± 2.2 Tg carbon per year, primarily due to increasing forest area. Temperature and precipitation increase the carbon density of woody debris, but decrease that of litter. Additionally, the woody debris increases significantly with above ground biomass and forest age. Our results can improve estimates of the carbon budget in China's forests and for better understanding of effects of climate and stand characteristics on dead organic matter distribution.

## Abstract (web summary)

Reliable estimates of the total forest carbon (C) pool are lacking due to insufficient information on dead organic matter (DOM). Here, the authors estimate that the current DOM C stock in China is 925 ± 54 Tg and that it grew by 6.7 ± 2.2 Tg C/yr over the past two decades primarily due to increasing forest area

## Introduction

Forests cover ~4200 million hectares (Mha) of the world's land surface^1^, and 50–90% of the total annual carbon (C) flux of terrestrial ecosystems occurs at the interface between forests and the atmosphere^2^. As key components of forest ecosystems, woody debris (including fine woody debris (FWD), snags, and logs) and litter represent important C stocks and have a strong influence on the structures and C dynamics of forest ecosystems^3^. Compared with C stocks in live biomass or soil, the C stock in dead organic matter (DOM, including woody debris and litter) has not been included in analysis of the large-scale C budget of China's forests^4–6^ because of a lack of reliable data from a sufficient number of representative sampling sites^7^. Thus, comprehensive assessments of the full forest C budget and its components are relatively rare, though such analyses are critically important for understanding how forest ecosystems respond to climate change, disturbances, and land management.

In natural or unmanaged forests, the DOM C stock is determined by the balance between the input and output of DOM, which is affected by various factors. In general, above ground biomass (AGB, including leaves, branches, tree tops, or whole trees) that dies or falls to the forest floor determines the potential input rate of DOM^8, 9^. Other factors, such as forest productivity, canopy cover, climate, and disturbance, also influence the input and output (decomposition) rates of DOM^10^. In addition, forest age is an important factor because it determines the accumulation of biomass and coarse woody debris (CWD, including snags and logs) stocks in forest ecosystems at a local scale^11, 12^.

Few studies have focused on the relationships between the driving factors and C stocks of DOM at the regional scale, except some work has been performed at the local scale^11–13^. Only a few country-scale studies have assessed the impacts of various driving forces on DOM C stocks^7, 14, 15^. Characterizing the relationships between potential driving factors and DOM C stocks is thus essential for understanding the current DOM C stock and for predicting the future evolution of ecosystem C stocks under different climate change scenarios.

Forests in China range from boreal forests in the northeast to tropical rain forests in the south and southwest, and include almost all of the major forest biomes in the Northern Hemisphere^16^. Forests are diverse in their species composition and climate conditions, thus providing unique venues for examining the spatial variations of DOM C density (C stock per unit area) and how it is impacted by climatic and biological factors. Although several studies have estimated C stocks of live biomass in China's forests^4, 17–19^, reliable estimates of DOM C stocks are still unavailable for this large and dynamic region. Therefore, a field-based estimate of the C stock of the entire forest sector in China is urgently needed to fulfill information needs that support management and policy decisions.

In this study, we documented estimates of DOM C stocks and changes in China's forests over the past two decades. We also examined the potential biotic and abiotic drivers that determined the spatial distribution of woody debris and litter. Specified regional Random Forest (RF) models for all DOM components were used to estimate the current distribution of DOM C and its change rates over the study period. We showed that the current DOM C stock in China's forests was 925 ± 54 Tg (5.95 ± 0.35 Mg C ha^−1^), with 429 ± 30 Tg in woody debris and 496 ± 24 Tg in litter. Live biomass was the dominant factor shaping the spatial distribution of the woody debris C, while climate determined the regional variation of the litter C stock. The DOM C stock increased by 6.7 ± 2.2 Tg C per year over the past 20 years, primarily due to increasing forest area.

## Results

### DOM C estimates

**Fig. 1:**
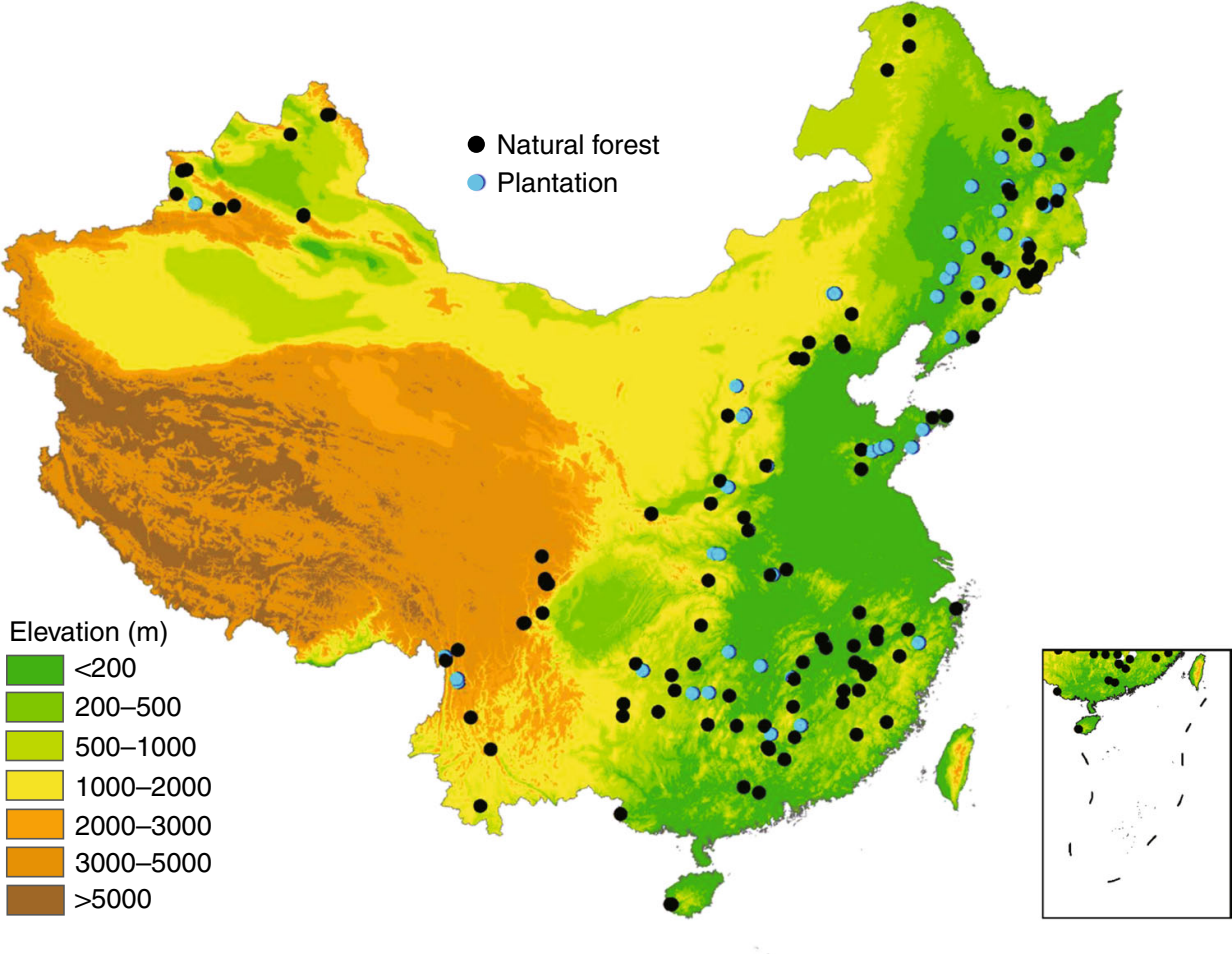
Distribution of 189 sampling sites in this study. The sites include 128 natural forests (65 primary forests and 63 secondary forests), and 61 plantations. For details, see Supplementary Data 1

**Fig. 2:**
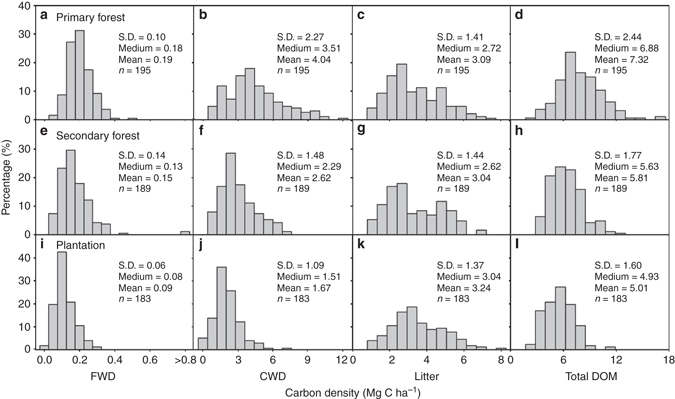
Frequency distribution of the carbon density of each component of dead organic matter (*DOM*). Frequency distributions are shown for **a**–**d** the primary forests, **e**–**h** secondary forests, and **i**–**l** plantations. Components of dead organic matter**d**, **h** and **l** include fine woody debris (*FWD*; **a**, **e** and **i**), coarse woody debris (*CWD*; **b**, **f** and **j**), and litter **c**, **g** and **k**

To quantify DOM C stocks in China's forests, a consistent field sampling protocol was used to investigate the C stocks of AGB, soil, woody debris, and litter from 567 field plots (20 × 20 m^2^) at 189 forest sites across the major forest types and climatic zones in China (Fig. 1, Supplementary Fig. 1, and Supplementary Data 1). The DOM C density in the sampled plots ranged from 2.0 to 16.2 Mg C ha^−1^ (1 Mg = 10^6^ g) with an overall mean of 6.1 ± 1.9 Mg C ha^−1^ (Fig. 2). The C density of the different DOM components was 0.0–1.3 Mg C ha^−1^ for FWD, 0.0–11.7 Mg C ha^−1^ for CWD (0.0–6.2 and 0.0–10.0 Mg C ha^−1^ for snags and logs, respectively), and 0.6–7.8 Mg C ha^−1^ for litter. The mean C density of DOM in different forest types ranged from 5.6 ± 1.9 Mg C ha^−1^ in conifer forests to 6.7 ± 1.7 Mg C ha^−1^ in evergreen broadleaf forests (Table 1). However, the C density of each DOM component varied substantially among the different forest types. For instance, the mean C density of woody debris was significantly lower in conifer forests (2.0 ± 1.4 Mg C ha^−1^) than in evergreen broadleaf forests (4.1 ± 1.6 Mg C ha^−1^) (*F* = 19.3, *P* < 0.001), but the mean C density of litter was significantly higher in conifer forests (3.5 ± 1.3 Mg C ha^−1^), and broadleaf and conifer mixed forests (3.6 ± 1.4 Mg C ha^−1^) than in evergreen broadleaf forests (2.0 ± 0.5 Mg C ha^−1^) (*F* = 12.1, *P* < 0.001; Table 1). For different origins of forests, primary forests had a significantly higher DOM C density (7.3 ± 2.4 Mg C ha^−1^) than secondary forests (5.8 ± 1.8 Mg C ha^−1^) and plantations (5.0 ± 1.6 Mg C ha^−1^) (*F* = 67.1, *P* < 0.001; Supplementary Fig. 2). However, the litter C density did not exhibit any significant differences among these three origins of forests (*F* = 1.1, *P* = 0.34).

**Table 1:** Area, number of sites, and carbon density of each carbon component for the different forest types^a^

Item	Evergreen broadleaf	Deciduous broadleaf	Broadleaf-conifer mixed	Conifer	Total
Area (Mha)	20.2	48.6	2.2	82.0	153.0
Number of sites	29	62	33	65	189
DOM (Mg C ha^−1^)	6.7 ± 1.7a	6.2 ± 1.9ab	6.4 ± 1.9a	5.6 ± 1.9b	6.1 ± 1.9
Woody debris	4.7 ± 1.6a	3.2 ± 1.8b	2.8 ± 1.5b	2.0 ± 1.4c	3.0 ± 1.8
FWD	0.22 ± 0.07a	0.15 ± 0.09b	0.15 ± 0.16b	0.12 ± 0.06b	0.15 ± 0.10
CWD	4.5 ± 1.6a	3.0 ± 1.7b	2.7 ± 1.4b	1.9 ± 1.3c	2.8 ± 1.7
Snags	2.0 ± 0.7a	1.6 ± 1.0b	1.2 ± 0.6c	0.9 ± 0.6c	1.4 ± 0.9
Logs	2.4 ± 1.1a	1.4 ± 0.9b	1.5 ± 1.0b	1.0 ± 0.9c	1.4 ± 1.0
Litter	2.0 ± 0.5c	3.0 ± 1.2b	3.6 ± 1.4a	3.5 ± 1.3a	3.1 ± 1.3
AGB (Mg C ha^−1^)	104.5 ± 51.3a	62.1 ± 34.7b	60.5 ± 30.2b	50.9 ± 21.8b	64.5 ± 37.6
Soil (Mg C ha^−1^)	72.2 ± 25.7a	71.6 ± 27.0a	67.7 ± 26.7a	63.1 ± 25.3a	68.1 ± 26.3
Ecosystem (Mg C ha^−1^)	183.5 ± 59.2a	140.4 ± 48.1b	134.5 ± 46.0bc	119.5 ± 31.8c	138.8 ± 49.2

*AGB* above ground biomass, *CWD* coarse woody debris, *DOM* dead organic matter, *FWD* fine woody debris

^a^The forest types are based on digitalized 1:1,000,000 vegetation map^55^. The data are presented as the means ± 1 SD, and the different letters (a, b, and c) denote significant differences at *P* < 0.05 across different forest types, via a one-way analysis of variance

Compared with the C density of AGB (64.5 ± 37.6 Mg C ha^−1^) and soil (68.1 ± 26.3 Mg C ha^−1^), the C density of DOM only accounted for 4.6 ± 1.5% of the total ecosystem C density (not including below ground biomass in this study) in the sampled sites, with 2.5 ± 1.4 and 2.1 ± 1.0% in litter and woody debris, respectively (Supplementary Fig. 3). The ratio of litter to total ecosystem C density was significantly different among the different forest types (*F* = 17.0, *P* < 0.001); the highest was in conifer forests (3.1 ± 1.3%) and the lowest was in evergreen broadleaf forests (1.2 ± 0.6%). However, the ratio of woody debris to total ecosystem C density was significantly lower in conifers (1.7 ± 1.0%) and broadleaf-conifer mixed forests (2.0 ± 0.8%) compared with that in deciduous broadleaf forests (2.2 ± 1.1%) and evergreen broadleaf forests (2.6 ± 0.7%) (*F* = 6.5, *P* < 0.001; Supplementary Fig. 3). The ratio of woody debris to total ecosystem C density in primary and secondary forests was similar (2.5 ± 1.1 vs. 2.2 ± 0.9%), and it was significantly higher than that in plantations (1.6 ± 0.8%) (*F* = 13.6, *P* < 0.001; Supplementary Fig. 3).

### Impacts of biotic and climatic factors on DOM C density

**Fig. 3:**
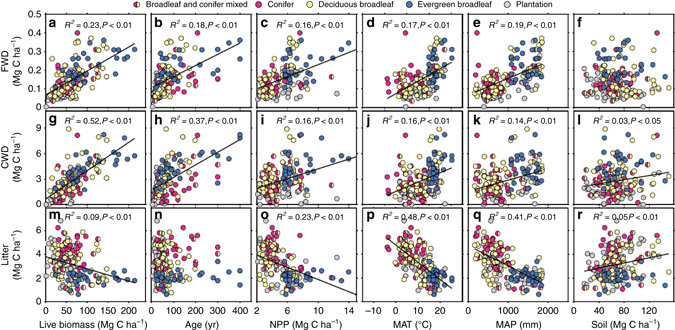
Relationships between carbon density of dead organic matter, and biotic and climatic factors. The *y* axis is carbon density (Mg C ha^−1^) of **a**–**f** fine woody debris (*FWD*), **g**–**l** coarse woody debris (*CWD*), and **m**–**r** litter. All plots showed the linear relationships between **a**, **g** and **m** above ground biomass carbon density, **b**, **h** and **n** forest age, **c**, **i** and **o** net primary production (NPP), **d**, **j** and **p** mean annual temperature (*MAT*), and **e**, **k** and **q** precipitation (*MAP*), and (**f**, **l** and **r**) soil carbon density and carbon density of each component of dead organic matter. There were no significant linear relationships between carbon density of litter and forest age, and between carbon density of FWD and carbon density of soil

Both FWD and CWD C densities were significantly positively correlated with AGB, forest age, and net primary production (NPP), but litter C density was negatively correlated with these factors (Fig. 3). Similarly, climatic factors, indicated by mean annual temperature (MAT) and precipitation (MAP), had significant positive effects on the C density of FWD and CWD (*P* < 0.001) but negative effects on the C density of litter (*P* < 0.001) (Fig. 3). In addition, the soil C density was positively correlated with the C density of CWD and litter (*P* < 0.05), but not withFWD.

### DOM C stocks in different inventory periods

Because there are significant correlations between the C density of each DOM component, and climate and stand characteristics (Fig. 3), we used these biotic and abiotic variables as predictors to estimate the spatial and temporal distributions of C density for each DOM component across China's forests. On the basis of the forest inventory and climatic data for the different inventory periods, from 1984−1988 to 2004−2008, we first used a specified regional RF model for each DOM component to map the grid-cell (at a resolution of 0.083°) C density of each DOM component across China's forest area for each inventory period (Supplementary Fig. 4). Then, we estimated the corresponding C density and C stock of each DOM component for each region and the whole country using the forest area for each inventory period (Table 2, Supplementary Table 1, see Methods).

**Table 2:** Modeled carbon density and stock of the forest dead organic matter in China for the forest inventory periods of 1984–1988 and 2004–2008. For information about the carbon density and stock during the other inventory periods, see Supplementary Table 1

C sector	Density (Mg C ha^−1^)		Stock (Tg C)	
	1984–1988	2004–2008	1984–1988	2004–2008
Woody debris	2.61 ± 0.18	2.76 ± 0.19	344 ± 24	429 ± 30
FWD	0.14 ± 0.01	0.14 ± 0.01	17.8 ± 1.3	21.6 ± 1.5
CWD	2.48 ± 0.18	2.61 ± 0.19	326 ± 24	407 ± 29
Snags	1.22 ± 0.11	1.31 ± 0.12	161 ± 14	203 ± 18
Logs	1.25 ± 0.10	1.31 ± 0.11	165 ± 13	204 ± 16
Litter	3.26 ± 0.17	3.19 ± 0.15	430 ± 22	496 ± 24
Total DOM	5.88 ± 0.35	5.95 ± 0.35	774 ± 46	925 ± 54

*CWD* coarse woody debris, *DOM* dead organic matter, *FWD* fine woody debris

**Fig. 4:**
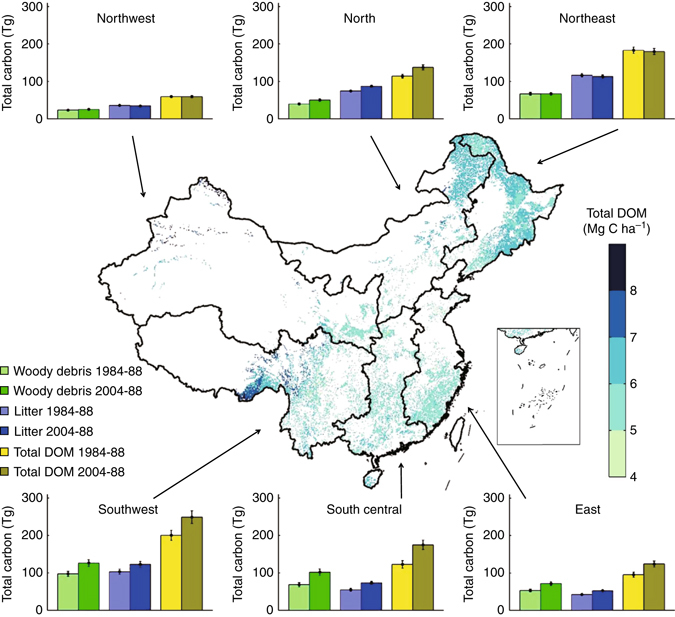
Carbon stocks of the woody debris and litter in the different regions of China from periods 1984–1988 to 2004–2008. The *filled colors* represent the model-based estimates of dead organic matter carbon density. Carbon stocks are presented as means  ± 1 SD, generated by 500 resamplings. Forests in China are divided into six regions: Northwest, including the provinces of Gansu, Ningxia, Qinghai, Shaanxi, and Xinjiang; North, including the provinces of Hebei, Inner Mongol and Shanxi, and the cities of Beijing and Tianjin; Northeast, including Heilongjiang, Jilin, and Liaoning provinces; Southwest, including the provinces of Guizhou, Sichuan, Yunan and Tibet, and the city of Chongqing; South Central, including the provinces of Guangdong, Guangxi, Hainan, Henan, Hubei and Hunan; and East, including the provinces of Anhui, Fujian, Jiangsu, Jiangxi, Shandong and Zhejiang, and the city of Shanghai. It should be noted that due to the lack of data, forests in Hong Kong, Macao and Taiwan were not included in the current study

On the basis of these calculations, the current (2004−2008) national DOM C density averaged 5.95 ± 0.35 Mg C ha^−1^ and showed a small variation among the different regions, ranging from 5.53 ± 0.36 Mg C ha^−1^ in southeastern forests to 6.28 ± 0.31 Mg C ha^−1^ in northern forests. However, the C density among the DOM components differed significantly, with woody debris accounting for >50% of the total DOM C density in southwestern, south central, and southeastern forests, while litter accounted for >60% of the total DOM C density in northwestern, northern, and northeastern forests (Fig. 4). The current national DOM C stock was estimated at 925 ± 54 Tg C (1 Tg = 10^12^ g), of which 429 ± 30 Tg C was stored in woody debris and 496 ± 24 Tg C was stored in litter (Table 2). Geographically, the largest DOM C stock (249 ± 17 Tg C, 27%) was in southwestern forests, followed by northeastern (180 ± 8 Tg C, 19%), south central (175 ± 13 Tg C, 19%), northern (137 ± 7 Tg C, 15%), and southeast (123 ± 8 Tg C, 13%) forests. The smallest DOM C stock was in northwestern forests (59 ± 3 Tg C, 6%) (Fig. 4).

**Fig. 5:**
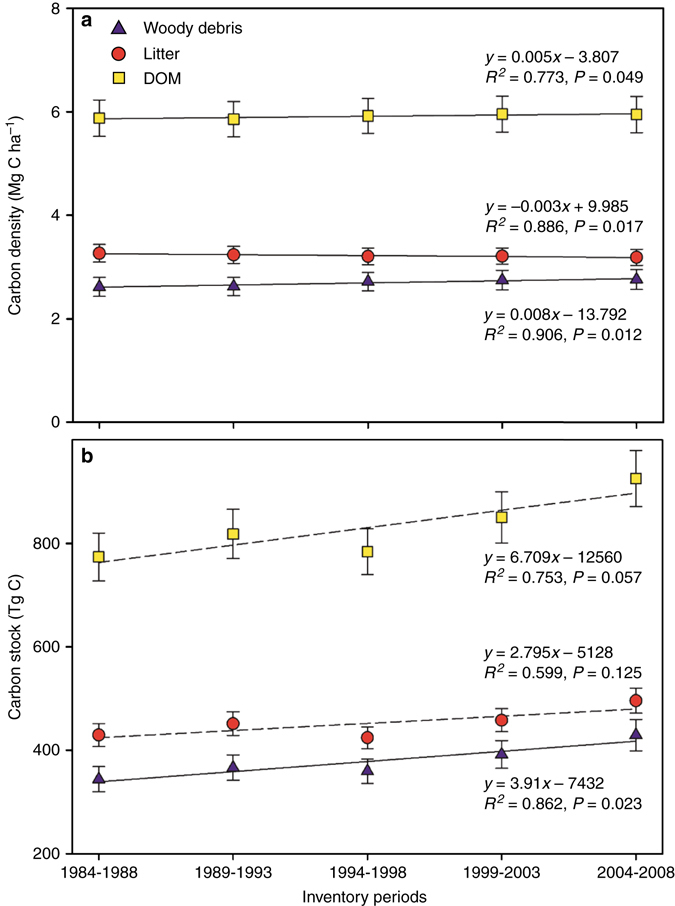
Changes in dead organic matter carbon over the past two decades. Changes in dead organic matter **a** carbon density and **b** carbon stock in China's forests from 1984–1988 to 2004–2008. Carbon densities and stocks are presented as means  ± 1 SD, generated by 500 resamplings. The fitted lines are from linear regression. Significant fitted lines (*P* < 0.05) are shown as the solid lines, and non-significant fitted lines (*P* > 0.05) are shown as the dotted lines

On the basis of the corresponding C density and C stock of each DOM component for each inventory period, we estimated their changes between 1984−1988 and 2004−2008 (Fig. 5). The C density of woody debris has significantly increased at a rate of 0.008 ± 0.002 Mg C ha^−1^ per year (~0.3% per year) over the past two decades (*R* ^2^ = 0.91, *P* = 0.01), whereas the C density of litter has significantly decreased at a rate of 0.003 ± 0.001 Mg C ha^−1^ per year (~0.1% per year; *R* ^2^ = 0.87, *P* = 0.02). As a result, the total DOM C density has increased by 0.005 ± 0.002 Mg C ha^−1^ per year (~0.1% per year; *R* ^2^ = 0.77, *P* < 0.05) over the two decades (Fig. 5a). Geographically, the largest increase in DOM C density (0.020 ± 0.007 Mg C ha^−1^ per year) occurred in the northern region with DOM C density increasing at a rate of 0.014 ± 0.004 Mg C ha^−1^ per year in woody debris and 0.006 ± 0.004 Mg C ha^−1^ per year in litter (Supplementary Fig. 5). Consistent with DOM C density, the national DOM C stock has continuously increased at a rate of 6.7 ± 2.2 Tg C per year (0.9% per year) through the past 20 years (Fig. 5b), primarily due to an increase in forest area (1.2 Mha per year, 0.9% per year; Supplementary Table 1). In total, 58% (3.9 ± 0.9 Tg C per year) of the increase in DOM C stock was attributed to woody debris and 42% (2.8 ± 1.3 Tg C per year) to litter.

## Discussion

Previous studies have investigated the spatial patterns of C stocks in live biomass and soils in China's forests^4, 5, 20^, but few studies have estimated the C stocks of full forest ecosystem components due to the lack of information on woody debris and litter. In this study, we quantified the C stock in woody debris and litter, as well as in AGB and soil in China's forests, to reduce uncertainties when assessing the C budget for forest ecosystem^5, 14^. Our estimates of the C density of woody debris (2.61 ± 0.18 Mg C ha^−1^) and litter (3.26 ± 0.17 Mg C ha^−1^) are very different particularly in components to those reported in Pan et al.^21^ (0.6 and 7.7 Mg C ha^−1^). These differences may be because their analyses were simply based on the data of live biomass of China's forest inventory reports that lacked direct field data on woody debris and litter.

**Fig. 6:**
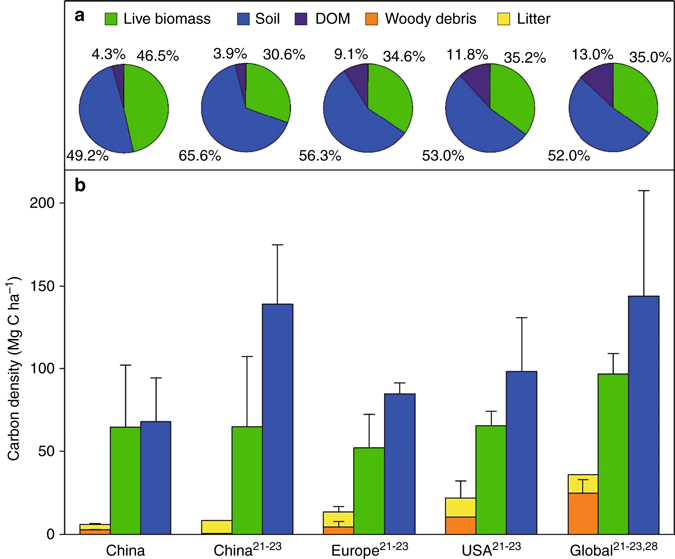
Carbon density of each forest ecosystem component in China compared with the values reported in other regions. **a** The pie charts show the contribution (%) of dead organic matter, live biomass and soil to total ecosystem carbon stock. **b** Carbon densities (Mg C ha^−1^) of the woody debris, litter, live biomass, and soil in China and other regions. The carbon density of Europe (geographic Europe), the United States, and the world are based on data reported in refs. ^21–23, 28^. Carbon densities in this study are presented as means  ± 1 SD, generated by 500 resamplings, and those in other studies are presented as means ± 1 SD from their reports (*n* = 3 for China, Europe and the USA; *n* = 4 for the world)

Compared with temperate forests in other regions, the DOM C density is relatively low in China (Fig. 6). For instance, our estimate of 5.88 ± 0.35 Mg C ha^−1^ was less than half that in the U.S. (15–29 Mg C ha^−1^) and Europe (11–16 Mg C ha^−1^)^21–23^. The low DOM C density may be caused by low input rates and/or high decomposition rates. Started in the later 1970s, China's reforestation and afforestation programs have led to the large area of young-growth plantations with less than 40 years old in China (~40 Mha, 26% of total forest area)^24^, which is more than 10 times of that in USA (3 Mha) and Europe (1 Mha)^23^. This large area of young-aged plantations and excessive harvest occurred before the reforestation programs have lower densities of live biomass and relatively shorter time of the accumulation of woody debris, and therefore cause a lower DOM C input^17, 24, 25^. In addition, it does not exclude the fact that DOM investigations in other countries tended to represent older, undisturbed forests with large woody debris stocks^21^ or forests that were recently affected by catastrophic disturbances^26, 27^, which resulted in higher estimates of the detrital C stock.

Comparing to the overall mean C density of AGB and soil in China's forests (i.e. 64.5 ± 37.6 and 68.1 ± 26.3 Mg C ha^−1^, respectively) (Table 1 and Fig. 6), the overall proportion of DOM C (6.1 ± 1.9 Mg C ha^−1^) was fairly small since being only 4.6 ± 1.5% of the entire ecosystem C, which is comparable to that reported by Pan et al.^21^ (5.4%) for China's forests but much lower than that in other temperate forests (8–18%)^21–23, 28^. Our current estimate was also confirmed by several other studies that have investigated the proportion of DOM C at the local scale: 4.4% in a subtropical evergreen broadleaf forest^29^, 4.0% in temperate deciduous broadleaf forests^30^, and 4.4% in a boreal forest^31^ in China. The small proportion of DOM C stock is likely a result of younger forests in China, which have lower DOM C stock and also incline to exempt intensive disturbances (e.g., wildfire), compared to temperate forests in other regions. In addition, the ratio of woody debris to AGB was small (4.9 ± 2.7%), much lower than that reported by Harmon and Hua^32^ (20–25% for subtropical and temperate forests). Woody debris only accounted for 2.1 ± 1.0% of the total C in China's forests, comparing to other temperate forests (4–18%) in the Northern Hemisphere^33^. It suggests that using empirical ratios of the global data would greatly overestimate woody debris C stock in China's forests.

Next, we discuss how stand characteristics and climatic factor influence DOM C density. In general, the rates of input (biomass production) and output (decomposition) determine the C density of woody debris and litter in natural or unmanaged forests. However, because the C of woody debris is persistent and slow to break down, its input rates ultimately outpace the decomposition rates. We found that woody debris was positively correlated with AGB, consistent with the finding from US forests in which the spatial distribution of downed woody debris was directly determined by the amount of live biomass^14^. Forest age was also expected to affect the amount of woody debris at the local scale, and often a greater amount of woody debris is found in young or old growth stands than in other stands (‘U- shaped’ pattern)^34^. However, our study suggests a monotonic increase of woody debris C density with forest age at the regional scale (Fig. 3). A study of forests in the US also reported similar results^14^. High stocks of woody debris commonly occur in mature forests that are highly stocked with live biomass^35^.

Besides the significant effect of stand characteristics, MAT and MAP also influenced positively FWD and CWD C density, although a US study found a negative trend in forests^7^. If climatic factors drive the distribution of forest live biomass^36, 37^, the potential amount of biomass input ultimately determines the spatial distribution of woody debris^38, 39^. The factor importance analysis (see Supplementary Materials and Methods) indicated that AGB was the optimal factor for predicting the spatial distribution of C density in all woody debris components (Supplementary Fig. 6). In contrast to the woody debris distribution, MAT and MAP were negatively correlated with litter distribution, which was likely related to faster losses of litter under hotter and wetter climates, which speed decomposition and turnover time^40^.

How much C has accumulated in China’s forest DOM over the past two decades? As shown in Fig. 5, the DOM density has increased by 0.005 ± 0.002 Mg C ha^−1^ per year in China's forests over the study period. The enhanced C accumulation in vegetation biomass in China's forests^4, 24, 41^ could increase potential DOM C inputs. Environmental changes, including increases in annual temperature and summer precipitation may contribute to the DOM C inputs because they could stimulate forest biomass C gain^42^, but they could also cause DOM C loss as a result of increased decomposition rate, especially of litter. In addition, large-scale reforestation and afforestation practices since the late 1970s have led to a significant increase in the forest area from 131.7 Mha in 1984–1988 to 155.6 Mha in 2004–2008, with an expansion rate of 0.9% per year. Compared with the small increased rate of DOM C density (0.1% per year), the expanding forest area (Supplementary Table 1) might be a dominant contributor to the accumulation in DOM C stocks for China's forests (Fig. 5).

According to previous estimates, the C sinks of forest biomass^24^ and soils^20^ over the past two decades were 70.9 and 67.2 Tg C per year, respectively. Together with the estimated C sink of 6.7 ± 2.2 Tg C per year in DOM in the current study, China's forests could have sequestered a total of ~ 145 Tg C per year from the 1980s to 2000s, which is roughly equivalent to 15.5% of the contemporary (1986–2006) fossil CO_2_ emissions in the country^43^.

Finally, we evaluated the uncertainty of the estimated C density of each DOM component at both the plot and pixel levels. At the plot level, the coefficient of determination (*R* ^2^) and root mean square error (RMSE) for each DOM component were calculated from the validation plot measurements (Supplementary Table 2). The averaged *R* ^2^ for woody debris and litter was 0.53 and 0.46, and the averaged RMSE for these components was 1.21 and 0.95 Mg C ha^−1^, respectively. At the pixel-scale, spatial distribution of the standard deviation (SD) for each DOM component was calculated using a Monte Carlo simulation (Supplementary Fig. 7).

Several uncertainties of our estimates of C stocks in woody debris, litter, AGB, and soil in China's forests must be acknowledged. First, relative to the large forest area (153 Mha) in China, the relatively small number of observed forest sites (*n* = 189 with 567 plots) could lead to imprecise estimates, although we put substantial effort to ensure high-quality data from field measurements representing the main forest biomes of China. Second, estimates excluded areas with logging residue piles from logging camps in China, which could result in an underestimation of national woody debris C stock. Lastly, seasonal variability could lead to uncertainty in the measurement of litter C stock. These biases in the field survey could have consequences of either an overestimation or an underestimation of DOM C density and may have introduced uncertainties in our results.

In conclusion, our study is the first national estimation of the C stock and the spatial distribution of DOM C density in China's forests. Our results indicate that the total DOM C stock in China's forests was 925 ± 54 Tg C (the area-weighted mean of 5.95 ± 0.35 Mg C ha^−1^), with 429 ± 30 Tg C in woody debris and 496 ± 24 Tg C in litter. The estimated DOM C stock showed a net gain of 6.7 ± 2.2 Tg C per year over the past 20 years. However, the ratio of DOM C to the overall forest C was as low as 4.6 ± 1.5%. With the ongoing national reforestation and afforestation programs in China, the forest DOM C stock is likely to continue to increase and should positively contribute to the entire forest C sink in the future.

## Methods

### Site description

The C stocks of all ecosystem C sectors (AGB, soil organic matter, woody debris, and litter) in China's forests were estimated from 567 sampling plots with an area of 20 × 20 m^2^at 189 forest sites (three replicated plots at each site). These forest sites range from 18.7 to 52.8 °N in latitude, from 81.0 to 131.2 °E in longitude, and from 30 to 3846 m above sea level, and represent almost all forest types in China, from the tropical rain forests in Hainan Island and Xishuangbanna to the boreal forests in the Greater Khingan Mountains (Fig. 1). MAT varies from −3.8 to 25.7 °C, and MAP ranges from 163 to 1850 mm across these sites, which almost covers the whole climate space in the country (Supplementary Fig. 1). Among these sites, there are 65 primary forests, 63 secondary forests, and 61 plantations (Supplementary Data 1).

### Field investigation

The fieldwork was conducted between 2011 and 2016 (Supplementary Data 1). According to Harmon et al.^3^, DOM was divided into three categories: (1) CWD, including standing snags and fallen logs, and defined as dead wood with a diameter of ≥10 cm at the maximum section, (2) FWD, defined as dead wood with a diameter of 2–10 cm, and (3) litter (defined as dead wood with a diameter of <2 cm and leaf litter). All these DOM components were collected from each forest plot and weighed. Some dead wood could not be directly weighed (i.e., logs with a diameter of ≥25 cm diameter and snags). For those dead woods, we first numbered, marked, and measured the length and diameter of the middle and both ends for the fallen logs, and measured the height and diameter at breast height (1.3 m, DBH) for the snags. We then weighed three parts of the log (the middle and both ends, three 10–20 cm length cylinders) after oven drying at 85 °C to a constant weight^44^. Next, we applied the mean ratio of the oven-dried weight to the volumes of the three woody cylinders to the total volume of the wood that could not be directly weighed to estimate its biomass. We also extrapolated the ratio to other large woody debris of the same species and determined the decay status to estimate the biomass.

The degree of CWD decay was defined in the following four classes: (I) recently fallen logs with wood and bark intact; (II) initial sapwood decay, bark loss, or easily removed bark and sound heartwood; (III) bark loss, heartwood decay, and large branch loss with stubs present; and (IV) rotten heartwood^45, 46^. The biomass of large snags was also extrapolated using the ratio of the weight to the volume of fallen logs in class (I) or (II). In addition, the volumes of individual snags and logs were calculated according to the following Eq. (1):

1 $${\rm{Volume}} = \frac{{\pi {d^2}L}}{4}$$

where *d* is the average diameter (middle and both ends) of the logs or the DBH for the snags and *L* is the length of the logs or the height of the snags.

The litter survey was performed at five subplots (2 × 2 m^2^, a total of 15 subplots for each site), and all the collected fine litter (including fresh and semi-decomposed leaf litter), reproductive structures, fallen bark, small dead wood <2 cm diameter (at the widest part) and other dead plant material (e.g. fallen leaves) in the surface litter layer were weighed after oven drying at 65 °C to a constant weight. The C concentrations of litter and woody debris in the four decay classes are shown in Supplementary Table 3.

In addition, we measured the height and DBH of all trees with a DBH of ≥5 cm in each plot. At each site, the ages of the 10 trees with the largest DBH were determined by performing a tree-ring analysis^47^. The age of the fifth-largest tree (DBH) at each site was used as the representative of the forest stand age^48^. The AGB of each tree and each plot was calculated using allometric equations (Supplementary Table 4) in relation to biomass components (leaf, bark, branch, and stem) according to the DBH (cm) and height (m) by species and regions.

We also sampled 1692 replicated soil profiles from 564 plots (three soil profiles from each plot) to estimate soil C density at the 188 forest sites across China (note: the soil data were not available at the Shennongjia site). Soils were separately sampled at depths of 0–10, 10–20, 20–30, 30–50, 50–70, and 70–100 cm. The soil bulk density at each depth was estimated using a standard container (100 cm^3^, 50.5 mm in diameter and 50 mm in height). The soil gravimetric moisture was measured after oven drying for 48 h at 105 °C. The soil subsamples were air dried for 2 weeks at room temperature (~25 °C) after the removal of debris and plant materials and then ground and sieved (0.15 mm) for C analysis. The C concentrations of DOM and soil were determined using an Elemental Analyzer (2400 II CHN Elemental Analyzer; Perkin-Elmer, Boston, MA, USA), and a factor of 0.5 was used to convert the live biomass to C^49, 50^. Thus, we obtained the C density of AGB, soil, FWD, CWD (snags and logs), and litter for each of the investigated sites (shown in Supplementary Data 1).

### Biomass and climate data

National forest inventory data were obtained to determine the forest area and timber volume for each forest type for each province. The inventory data in five periods of 1984–1988, 1989–1993, 1994–1998, 1999–2003, and 2004–2008 were used in this study^51–55^. The following previously determined biomass expansion factor (ratio of biomass to volume) was used to convert forest timber volume to biomass^4, 5, 24, 50^:

2 $${\rm{BEF = }}a + \frac{b}{x}$$

where *x* is the timber volume per unit area (m^3^ ha^−1^ in this study) and *a* and *b* are constants for a specific forest type. We used Eq. (2) to calculate the provincial forest biomass for each forest type for each period based on the corresponding area and volume in the forest inventory data. Next, a factor of 0.5 was used to convert the biomass to C^49, 50^.

The digitized 1:1,000,000 vegetation map^56^ was used to obtain the spatial distribution of national vegetation for 161 forest types (excluding bamboo forests). Eighteen forest types defined from the forest inventory were matched within the 161 forest types defined using the digitized vegetation map and then used for the calculations (Supplementary Table 5). The grid-cell AGB C density for each forest type in each province was the area-weighted averaged value, which was used to obtain forest biomass C density distribution during different inventory periods (Supplementary Fig. 8).

The monthly air temperature and precipitation data were obtained from 728 meteorological stations (National Meteorological Information Center of the China Meteorological Administration, http://data.cma.cn ^57^. We interpolated the climate data to grid cells with a resolution of 0.083° using a Kriging interpolation algorithm. We obtained MAT and MAP for each site and each period.

In addition, we documented mean net primary productivity (NPP) from a 10-year average (2000–2009) of a Moderate Resolution Imaging Spectroradiometer (MODIS) NPP product^58, 59^ for the analysis of input C to DOM stocks. The mean NPP is the summation of 8-day calculations of net photosynthesis minus maintenance and growth respiration and had a nominal 0.083°resolution. For MAT, MAP, and NPP data, we extracted site-specific values from dataset according to longitude and latitude of the sampling sites.

### Statistical Analyses

We constructed RF models^60^ to simulate the national grid-cell C density of woody debris (FWD, snags, and logs) and litter. The RF fitted multiple decision trees to input data using a random subset of the input variables for each tree constructed for each response variable. Four factors (MAT, MAP, AGB C density, and forest type) were used as input variables to predict the DOM C density. The data set of each DOM component was randomly divided into two parts: the training dataset (70% of all sites, 132 sites) and the validation data set (30% of all sites, 57 sites). The training data set along with the four predicted factors was used to estimate the C density of each DOM component. We then acquired the coefficient of determination (*R* ^2^) and root mean square error (RMSE) between the predicted and measured values from the validation data set in each re-sampling. The training and evaluation procedures were repeated 500 times for each DOM component. As a result, we acquired 500 results for model estimation and evaluation. The *R* ^2^ and RMSE in Supplementary Table 2 represent the average value of 500 model simulations. To evaluate the performance of the predicted factors, we explored the relative importance of each variable in a RF model by calculating the percentage increase in the mean square error (%IncMSE) (Supplementary Fig. 6). We then used a Monte Carlo approach to propagate the model errors to pixel-, regional-, and national-scale estimates. Next, we applied the 500 RF models to estimate the C density of all the DOM components for the periods of 1984–1988, 1989–1993, 1994–1998, 1999–2003, and 2004–2008 according to the historical forest types, biomass data (Supplementary Fig. 8), and the related MAT and MAP data. The means and standard deviations (SDs) of the grid-cell, regional, and national C density of each DOM component were generated by all the random models. The final predictions for each DOM component and each period were the average estimates of the 500 RF models. On the basis of the results of the RF models, the spatial distribution of the national FWD, snags, logs, CWD, woody debris, litter, and total DOM C density and their pixel-scale SDs were mapped at a resolution of 0.083°.

To capture the change rate of the DOM C density and stock for each component, we generated a simple linear regression between the C density/stock and the year (1986, 1991, 1996, 2001 and 2006). The slope of the regression and its SD were defined as the change rate and the SD for each DOM C density and stock. The observed data in this study are listed in Supplementary Data 1.

### Data availability

Basic information on locations, forest type, stand age, dominant tree species, and the data for C density of each forest sector of the study sites are listed in Supplementary Data 1. The input data (mean annual temperature and precipitation, forest type, and forest biomass) used can be found in the corresponding references. The remaining data that support the findings of this study can be requested from the corresponding author.

## Electronic supplementary material

Supplementary Information

Supplementary Data 1
