# Supplementary material for: Carbon stocks and changes of dead organic matter in China's forests
Source: Nat Commun. 2017 Jul 28;8:151. doi: 10.1038/s41467-017-00207-1 (PMC5532249; doi:10.1038/s41467-017-00207-1)
Supplement: Supplementary file 1 — Supplementary Information [file 41467_2017_207_MOESM1_ESM.pdf]

File name: Supplementary Information
Description: Supplementary Figures, Supplementary Tables and Supplementary References

File name: Supplementary Data 1
Description: The dataset of dead organic matter in China's forests. It includes 422 the following information: locations, forest type, stand age, dominant tree species, and carbon density (Mg C ha-1 423) of aboveground biomass, soil and each dead organic matter component 424 for all sampling sites. The carbon density was an average of that of 3 plots for each site.

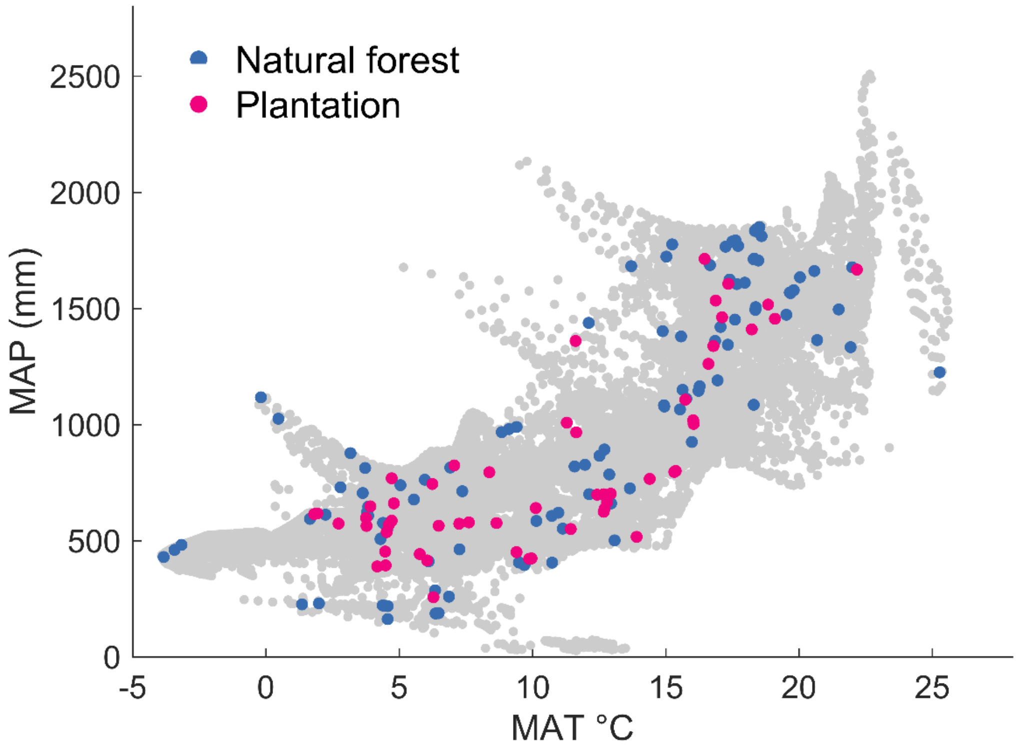

Supplementary Figure 1 | Distribution of sampling sites in climatic space. The gray areas represent the extent of forest distribution in China obtained from the digitalized 1:1,000,000 vegetation map[1].

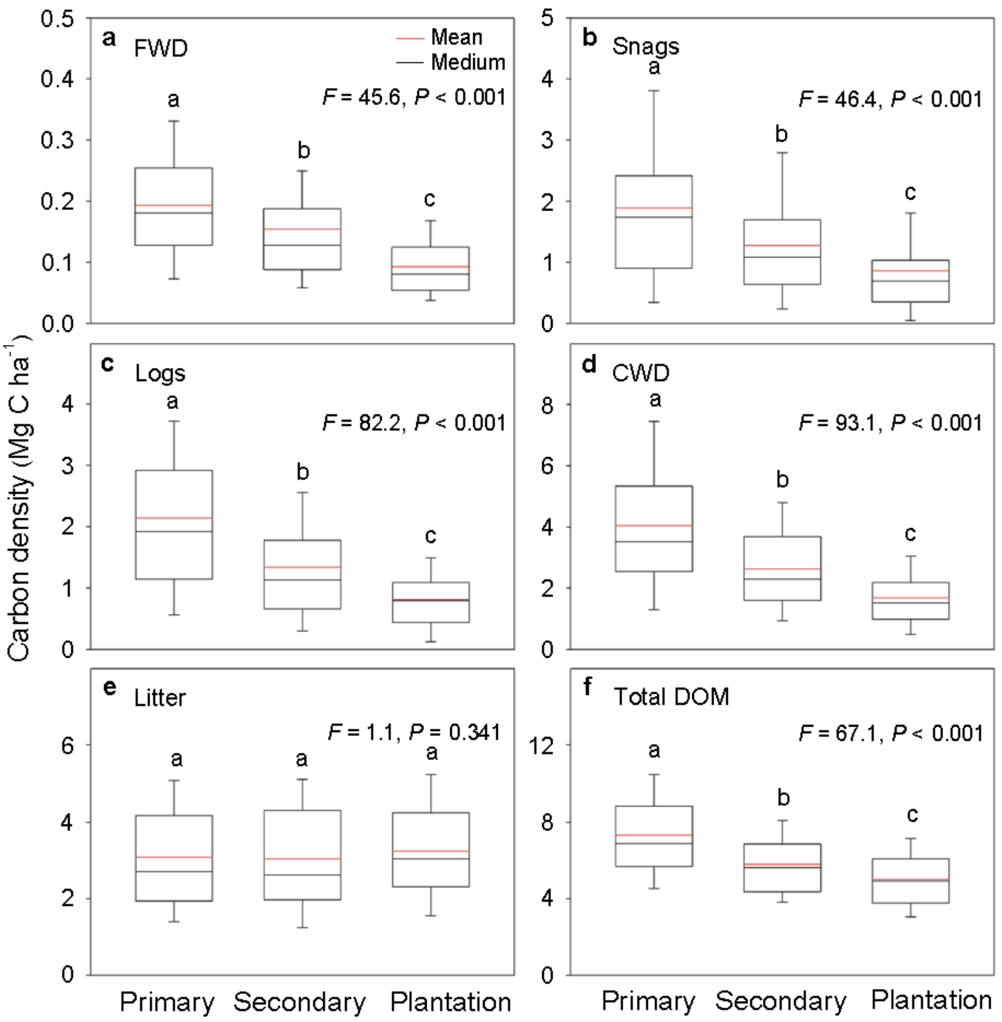

Supplementary Figure 2 | Carbon densities of different dead organic matter components in China's forests by forest origin. Forest origin includes the primary, secondary, and planted forests. (**a**) Fine woody debris (FWD), (**b**) snags, (**c**) logs, (**d**) coarse woody debris (CWD), (**e**) litter, and (**f**) total dead organic matter (DOM). Boxes in box plots extend from the first (25%) and third (75%) quartiles, with black line at the median and red line at the mean value, and whiskers extend from the 5th to the 95th percentile (n = 195 for the primary forests, n = 189 for the secondary forests, and n = 183 for the plantations). Different letters denote significant differences at $P < 0.05$, via a one-way analysis of variance.

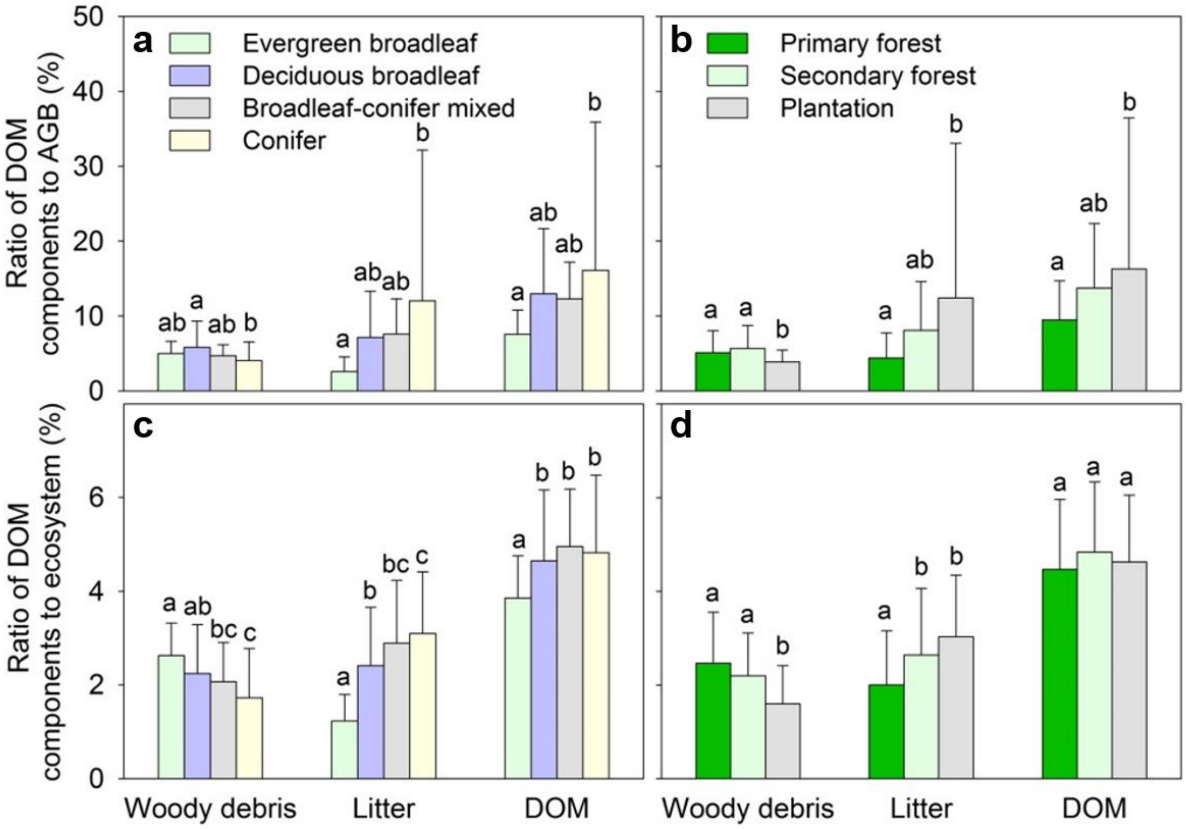

Supplementary Figure 3 | Ratios of dead organic matter carbon to aboveground biomass and the total ecosystem carbon. Ratios (%) of woody debris, litter and total dead organic matter (DOM) to (**a** and **b**) aboveground biomass (AGB) and (**c** and **d**) the total ecosystem carbon for (**a** and **c**) different forest types and (**b** and **d**) different forest origins. Data are presented as means ± 1 SD, n = 87, 186, 99 and 195 for evergreen broadleaf, deciduous broadleaf, broadleaf-conifer mixed and coniferous forests, respectively, and n = 195, 189 and 183 for the primary, secondary and planted forests, respectively. Different letters denote significant differences at $P < 0.05$, via a one-way analysis of variance.

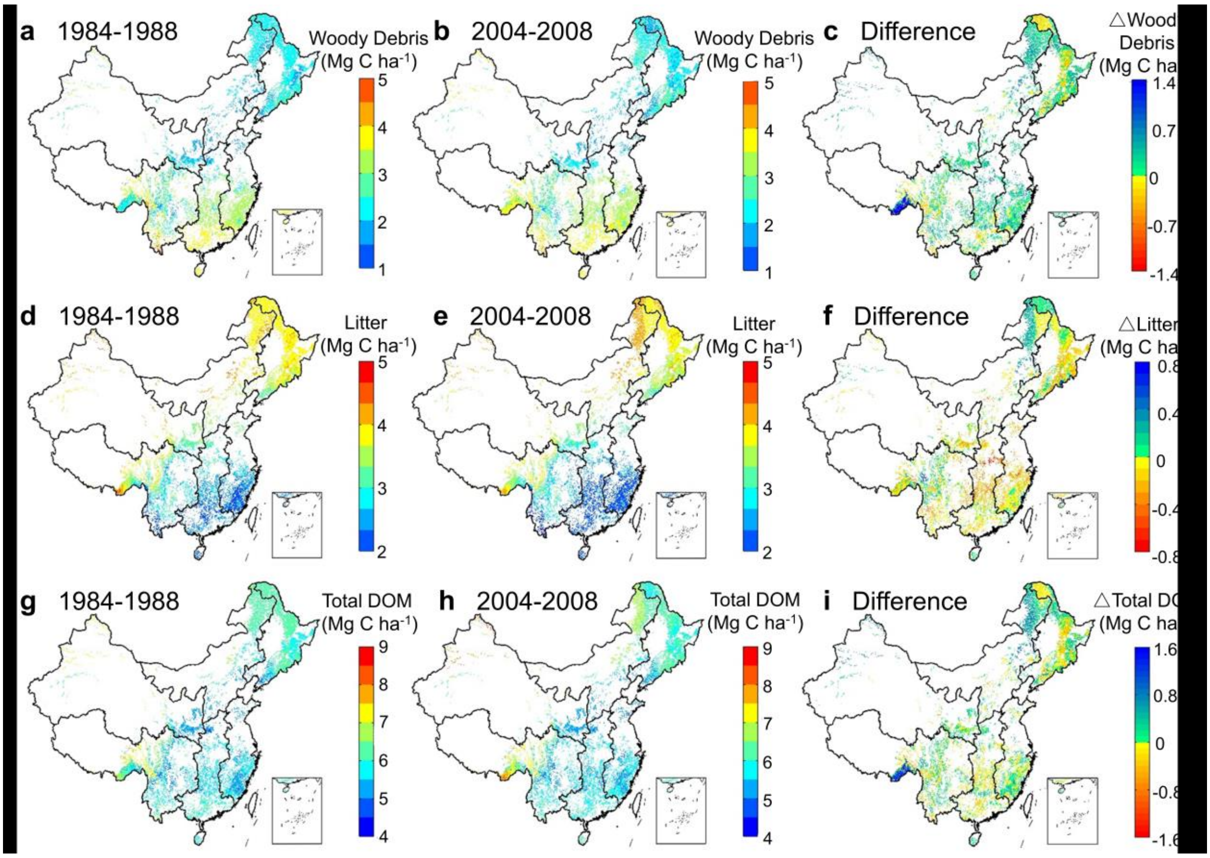

Supplementary Figure 4 | Spatial distributions and differences in carbon density of dead organic matter. Distributions in carbon densities (Mg C ha^{-1}) of (**a**-**c**) woody debris, (**d**-**f**) litter and (**g**-**i**) the total dead organic matter (DOM) for the inventory periods of (**a**, **d** and **g**) 1984–1988 and (**b**, **e** and **h**) 2004–2008 and (**c**, **f** and **i**) their differences in China's forests were estimated by 500 Random Forest models.

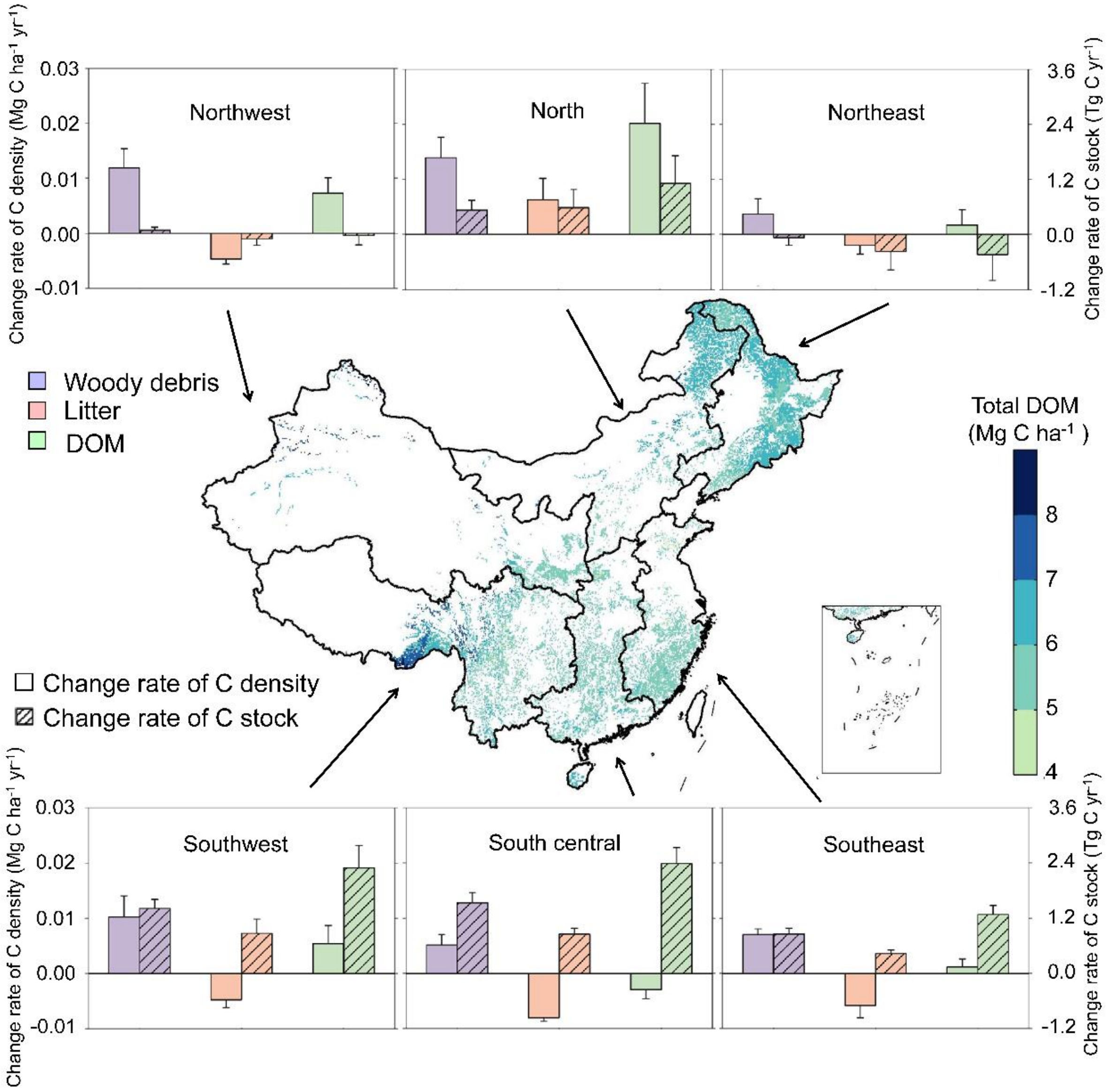

Supplementary Figure 5 | Change rates of carbon density and stock of dead organic matter. Change rates of carbon density (Mg C ha^{-1} yr^{-1}) and stock (Tg C yr^{-1}) of woody debris, litter, and total dead organic matter (DOM) in different regions of China from 1984–2008 are presented as means ± 1 SD, generated by 500 resamplings.

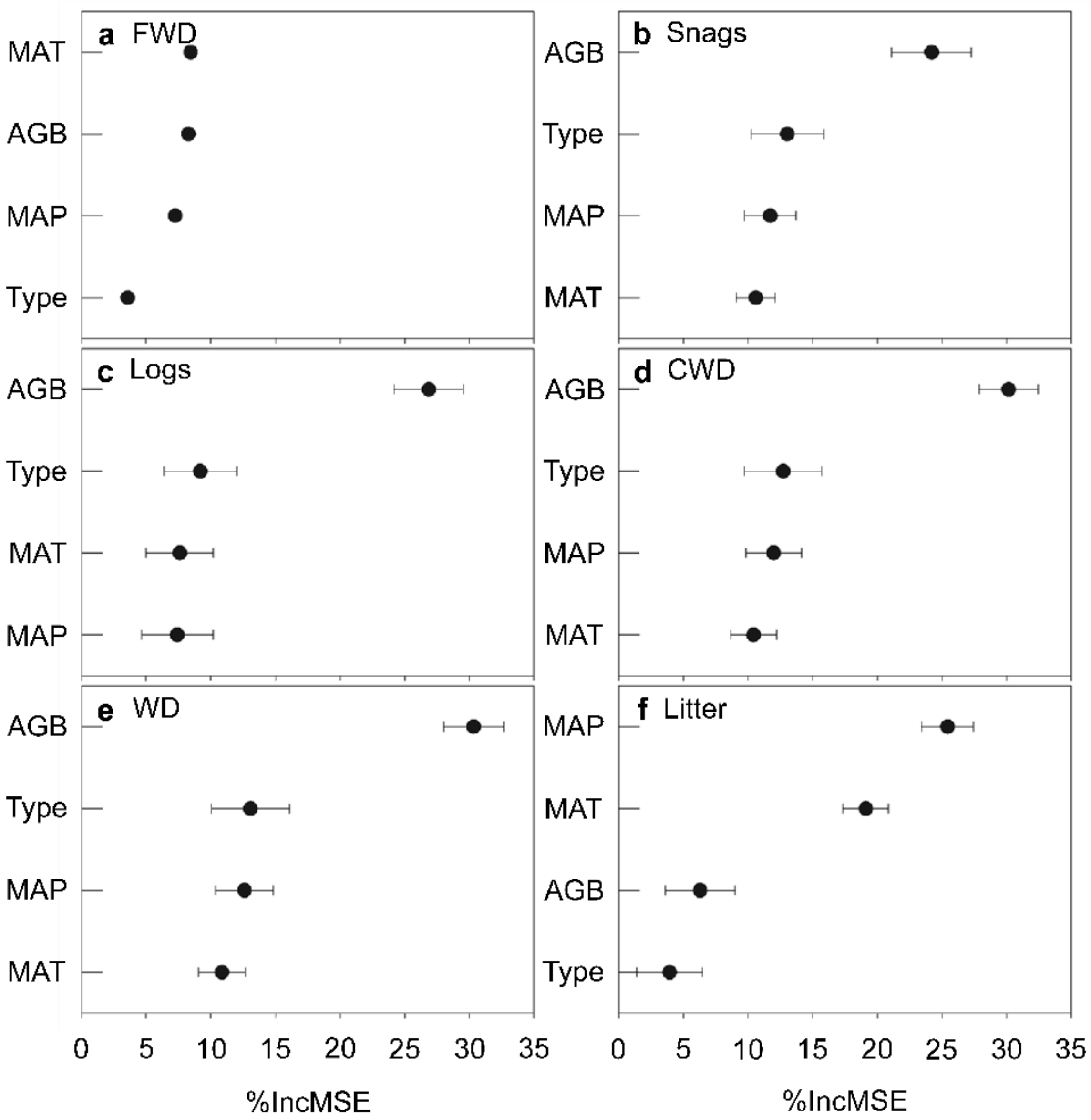

Supplementary Figure 6 | Importance of the variables in the Random Forest models built from a training dataset denoted by percentage increase of mean squared error. Values of the percentage increase of mean squared error (%IncMSE) are means ± 1 SD generated from 500 resamplings of the carbon density of (**a**) fine woody debris (FWD), (**b**) snags, (**c**) logs, (**d**) coarse woody debris (CWD), (**e**) woody debris (WD) and (**f**) litter. AGB, Type, MAT and MAP represent aboveground biomass carbon density, forest type, mean annual temperature and precipitation, respectively.

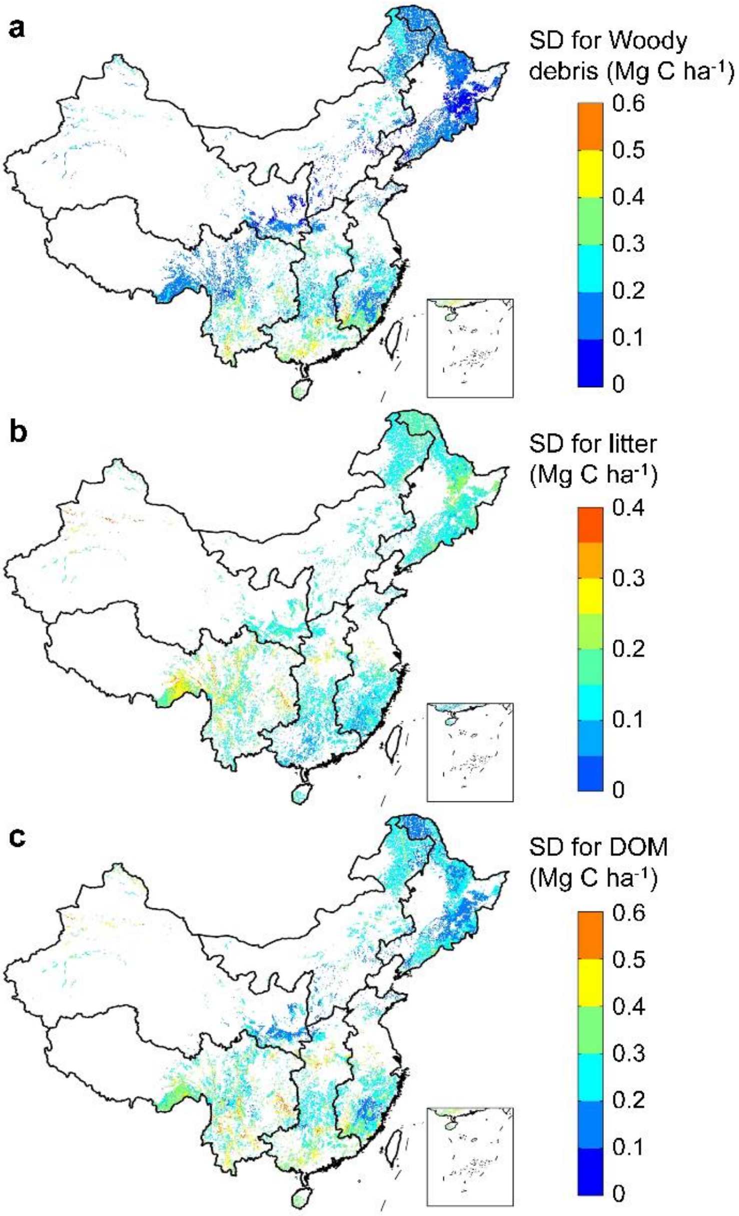

Supplementary Figure 7 | Spatial distributions of standard deviation for dead organic matter. The standard deviation (SD) of the grid-cell carbon density of (**a**) woody debris, (**b**) litter and (**c**) total dead organic matter (DOM) in China's forests were generated by 500 Random Forest models.

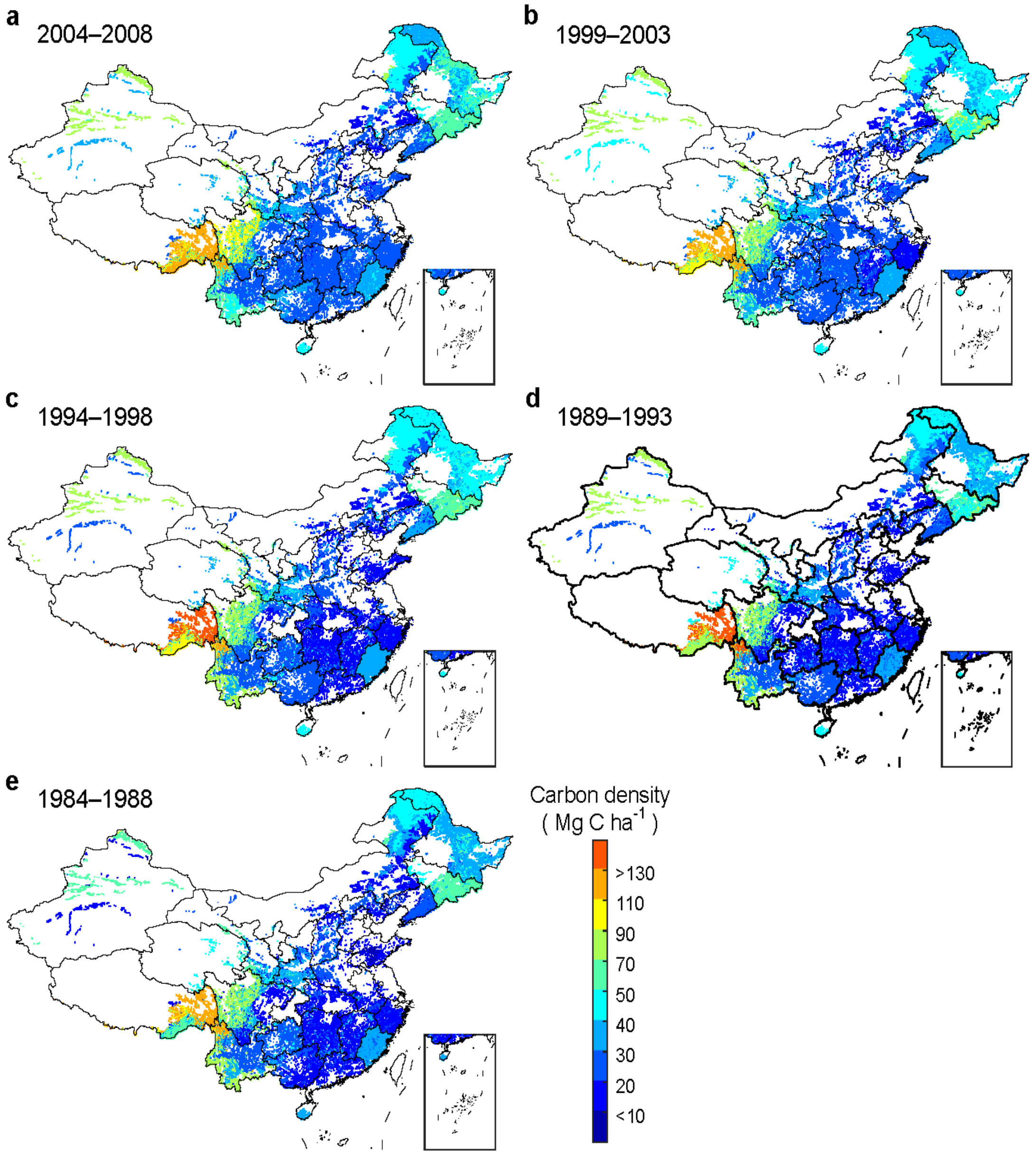

Supplementary Figure 8 | Spatial distribution of carbon density of aboveground biomass in China's forests in different inventory periods. (**a**) 2004–2008, (**b**) 1999–2003, (**c**) 1994–1998, (**d**) 1989–1993, and (**e**) 1984–1988.

Supplementary Table 1 | Modeled carbon density (Mg C ha^{-1}) and stock (Tg C) for dead organic matter in China's forests for five inventory periods of 1984–1988, 1989–1993, 1994–1998, 1999–2003, and 2004–2008.

C sector	1984–1988	1989–1993	1994–1998	1999–2003	2004–2008
Forest area (Mha)	131.7	139.7	132.4	142.8	155.6
Density (Mg C ha^{-1})					
Woody debris	2.61±0.18	2.62±0.18	2.72±0.18	2.74±0.19	2.76±0.19
FWD	0.14±0.01	0.14±0.01	0.14±0.01	0.14±0.01	0.14±0.01
CWD	2.48±0.18	2.49±0.17	2.57±0.17	2.60±0.18	2.61±0.19
Snags	1.22±0.11	1.23±0.10	1.27±0.10	1.30±0.11	1.31±0.12
Logs	1.25±0.10	1.25±0.10	1.30±0.10	1.31±0.11	1.31±0.11
Litter	3.26±0.17	3.23±0.16	3.20±0.16	3.21±0.16	3.19±0.15
**Total DOM**	**5.88±0.35**	**5.85±0.34**	**5.92±0.33**	**5.95±0.35**	**5.95±0.35**
Stock (Tg C)					
Woody debris	344±24	366±25	360±23	392±27	429±30
FWD	18±1	19±1	19±1	20±2	22±2
CWD	326±24	347±24	341±23	371±26	407±29
Snags	161±14	172±14	168±14	185±16	203±18
Logs	165±13	175±14	173±14	187±15	204±16
Litter	430±22	452±23	424±21	458±22	496±24
**Total DOM**	**774±46**	**818±47**	**784±44**	**850±49**	**925±54**

Supplementary Table 2 | Coefficient of determination (R^2) and root mean square error (RMSE, Mg C ha^{-1}) of the Random Forest models.

Model	R^2	RMSE
Woody debris	0.53	1.21
FWD	0.36	0.08
CWD	0.51	1.18
Snags	0.41	0.65
Logs	0.46	0.74
Litter	0.46	0.95

Notes: Values are the averaged R^2 and RMSE of 500 RF models. FWD and CWD represent fine woody debris and coarse woody debris, respectively.

Supplementary Table 3 | Carbon concentration (%) for litter and woody debris in different decay classes in six regions of China.

Region	Litter	Decay class for woody debris			
		1	2	3	4
Northeast	40.7±7.1	49.5±1.2	47.2±1.2	49.7±1.7	50.8±1.8
North	41.9±6.2	51.0±4.7	51.4±4.2	54.0±3.5	54.6±4.2
Northwest	41.7±5.1	45.7±0.6	45.7±0.5	46.1±0.5	47.6±1.4
East	42.0±7.8	43.9±1.3	42.7±3.2	44.7±2.2	45.9±1.9
South central	42.3±5.2	48.4±2.3	48.4±2.2	48.7±2.5	49.9±3.5
Southwest	41.9±6.6	47.3±4.2	48.0±4.1	48.1±4.1	48.8±3.8
Total	41.7±6.6	48.1±3.3	47.6±3.3	48.5±3.3	49.7±3.6

Supplementary Table 4 | Equations for calculating aboveground biomass (AGB, kg) of dominant tree species in different regions used in this study. D and H are diameter at breast height (cm), and height (m) of a tree, respectively.

Region	Species	Equation	Ref.
Northeast	*Abies fabri*	$AGB=exp(-2.943+logD^{2.608})$	2
	Abies nephrolepis	AGB=exp(-2.943+2.608ln(D))	2
	Acer mono	$AGB=0.3723D^2$	3
	Betula platyphylla	$AGB=10\hat{}(2.159+logD^{2.367})/1000$	4
	Fraxinus mandschurica	$AGB=0.4803D^2$	3
	Juglans mandshurica	$AGB=10\hat{}(2.235+logD^{2.287})/1000$	4
	Larix	$AGB=0.046238(D^2H)^{0.905002}$	5
	Phellodendron amurense	$AGB=10\hat{}(1.942+logD^{2.332})/1000$	4
	Picea asperata	$AGB=0.067732(D^2H)^{0.865949}$	5
	Pinus koraiensis	$AGB=10\hat{}(2.236+logD^{2.144})/1000$	4
	Pinus sylvestris	$AGB=0.2792D^2$	3
	Populus	$AGB=0.3521D^2$	3
	Quercus mongolica	$AGB=10\hat{}(2.002+logD^{2.456})/1000$	4
	Tilia mandshurica	$AGB=0.045H^{1.9768}$	6
	Ulmus	$AGB=0.3593D^2$	3
	Other *Acer*	$AGB=0.0786H^{1.8251}$	6
Northwest	*Abies*	$AGB=0.067732(D^2H)^{0.865949}$	5
	Betula	$AGB=0.14114(D^2H)^{0.7234}+0.00724(D^2H)^{1.0225}+0.01513(D^2H)^{0.8085}$	7
	Larix	$AGB=exp[0.7681ln(D^2H)-1.9164]\times4.81/5.81$	8
	Picea	$AGB=0.1244(D^2H)^{0.8052}$	9
	Pinus	$AGB=exp[0.9302ln(D^2H)-2.9132]\times3.14/4.14$	10
	Populus	$AGB=exp[0.9222ln(D^2H)-2.836]\times2.89/3.89$	10
	Quercus liena	$AGB=exp[0.9979ln(D^2H)-3.5426]\times3.85/4.85$	10
	Quercus mongolica and *Q. liaotungensis*	$AGB=0.0369(D^2H)^{0.9165}+0.00051(D^2H)^{1.3377}+0.00021(D^2H)^{1.171}$	11

	Quercus variabilis	$AGB=\exp[0.8579\ln(D^2H)-2.006]\times3.85/4.85$	10
	Other hardwood	$AGB=\exp[0.9037+\ln(D^2H)-2.57]\times3.85/4.85$	10
North	*Betula*	$AGB=0.03190(D^2H)^{0.9356}+0.00063(D^2H)^{1.2781}+0.00016(D^2H)^{1.1688}$	11
	Fraxinus	$AGB=0.6031(D^2H)^{0.8906}+0.00556(D^2H)^{1.169}+0.00829(D^2H)^{0.9919}$	12
	Juglans	$AGB=0.02511(D^2H)^{0.9271}+0.00957(D^2H)^{0.974}+0.08725(D^2H)^{0.2634}$	12
	Larix	$AGB=0.046238(D^2H)^{0.905002}$	5
	Pinus tabulaetormis	$AGB=0.0475(D^2H)^{0.8539}+0.0017(D^2H)^{1.1515}+0.0134(D^2H)^{0.8099}+0.0013(D^2H)^{0.9055}$	11
	Platycladns	$AGB=0.022857(D^2H)+0.12531(D^2H)^{0.733}+0.190893$	13
	Quercus	$AGB=0.0369(D^2H)^{0.9165}+0.00051(D^2H)^{1.3377}+0.00021(D^2H)^{1.171}$	11
	Ulmus	$AGB=0.05229(D^2H)^{0.891}+0.01233(D^2H)^{0.9359}+0.01736(D^2H)^{0.7738}$	12
East	*Castanopsis*	$AGB=0.06491(D^2H)^{0.92}$	14
	Cunninghamia lanceolata	$AGB=0.073429(D^2H)^{0.86262}+0.013775(D^2H)^{0.84463}+0.000482(D^2H)^{1.23314}+0.019638(D^2H)^{0.78969}$	5
	Cyclobalanopsis	$AGB=0.08542(D^2H)^{0.91}$	14
	Lithocarpus	$AGB=0.04268(D^2H)^{0.98}$	14
	Loropetalum	$AGB=0.2212D^{2.35119}$	14
	Pinus elliottii	$AGB=0.11083(D^2H)^{0.792665}$	15
	Pinus massoniana	$AGB=0.1377(D^2H)^{0.8172}\times6.23/7.23$	16
	Quercus	$AGB=0.1199(D^2H)^{0.8509}$	14
	Rhododendron	$AGB=0.2212D^{1.9932}$	14
	Schima superba	$AGB=0.031103(D^2H)^{1.019796}$	15
	Other species	$AGB=0.09459(D^2H)^{0.87}$	14
South Central	*Castanopsis*	$AGB=0.06491(D^2H)^{0.92}$	14
	Cinnamomum	$AGB=0.055603(D^2H)^{0.850193}+0.014757(D^2H)^{0.808395}+0.006652(D^2H)^{1.051841}+0.059871(D^2H)^{0.574327}$	17
	Cunninghamia lanceolata	$AGB=\exp(2.3919\ln D-2.52147)$	18
	Fagus	$AGB=0.0125(D^2H)^{1.05}+0.000933(D^2H)^{1.23}+0.000294(D^2H)^{1.20}$	19
	Pinus massoniana	$AGB=10^\wedge[4.44125\log(D^2H)-11.6149]$	20
	Populus	$AGB=10^\wedge[1.00110\log(D^2H)-1.62289]$	21
	Quercus	$AGB=0.3108(D^2H)^{0.67428}+0.0293(D^2H)^{0.75662}+0.0922(D^2H)^{0.39445}+0.93685(D^2H)^{0.614021}$	19
	Robinia	$AGB=0.0319(D^2H)^{0.9356}+0.00063(D^2H)^{1.2781}+0.00016(D^2H)^{1.1688}+0.01388(D^2H)^{0.8102}$	19

	Schima superba	$AGB=0.07103(D^2H)^{0.91}$	14
	Other temperate deciduous species	$AGB=0.082D^{2.38}+2.14D^{1.03}+0.277D^{0.93}$	22
	Other temperate evergreen species	$AGB=0.00707D^{3.32}+0.762D^{1.15}+0.314D^{1.12}$	22
	Other tropical species	$AGB=0.040213(D^2H)^{0.97268}$	23
	Other species	$AGB=0.09459(D^2H)^{0.87}$	14
Southwest	*Abies*	$AGB=0.0139(D^2H)^{1.0075}+0.0014(D^2H)^{1.0503}+0.0003(D^2H)^{1.2032}$	24
	Acer	$AGB=0.3274(D^2H)^{0.7218}+0.01349(D^2H)^{0.7198}+0.02347(D^2H)^{0.6929}$	25
	Alnus cremastogyne	$AGB=0.1565(D^2H)^{0.7388}$	26
	Betula	$AGB=0.14114(D^2H)^{0.7234}+0.00724(D^2H)^{1.0225}+0.01513(D^2H)^{0.8085}$	27
	Cupressus funebris	$AGB=0.2045(D^2H)^{0.7499}$	26
	Larix	$AGB=0.0204(D^2H)^{0.9719}$	28
	Picea	$AGB=3.1660(D^2H)^{0.4567}+0.8657(D^2H)^{0.4109}+12.4382(D^2H)^{0.1928}+2.9259(D^2H)^{0.3129}$	29
	Pinus massoniana	$AGB=0.0487(D^2H)^{0.9320}$	30
	Tsuga	$AGB=0.149707(D^2H)^{0.80139}$	5
	Other broadleaf species	$AGB=0.0152(D^2H)+10.0895$	24
Total	*Phyllostachys*	$AGB=0.6439D^{1.5373}$	5
	Other *Betula*	$AGB=0.0278601(D^2H)^{0.993386}$	5
	Other *Pinus*	$AGB=0.071556(D^2H)^{0.857209}$	5
	Other softwood	$AGB=0.0495502(D^2H)^{0.952453}$	5
	Other hardwood	$AGB=0.044(D^2H)^{0.9169}+0.0023(D^2H)^{0.7115}+0.0104(D^2H)^{0.994}+0.0188(D^2H)^{0.8024}$	5

Supplementary Table 5 | Forest type groups used in forest inventory correspond to the forest types in the digitized 1:1,000,000 vegetation map.

Major class	Forest type group in forest inventory	Forest types in vegetation map
Conifer	*Abies* and *Picea* forest	*A. delavayi* forest, *A. delavayi* var. *motuoensis* forest, *A. densa* and *P. spinulosa* mixed forest, *A. fabri* forest, *A. fargesii* forest, *A. faxoniana* forest, *A. forrestii* forest, *A. georgei* forest, *A. georgei* var. *smithii* forest, *A. kawakamii* forest, *A. nephrolepis* forest, *A. spectabilis* forest, *A. squamata* forest, *P. asperata* forest, *P. brachytyla* forest, *P. crassifolia* forest, *P. jezoensis* forest, *P. likiangensis* forest, *P. likiangensis* var. *balfouriana* forest, *P. schrenkiana* forest, *P. likiangensis* var. *linzhiensis* forest, *P. meyeri* forest, *P. purpurea* forest, and *P. wilsonii* forest
	Cypress forest	*Cupressus funebris* forest, *Platycladus orientalis* forest, *Sabina convallium* forest, *S. przewalskii* forest, *S. saltuaria* forest, *S. squamata* forest, *S. tibetica* forest, *S. komarovii* forest
	Cunnunghamia lanceolata forest	
	Larix forest	*L. gmelinii* forest, *L. kaempferi* forest, *L. olgensis* forest, *L. potaninii* forest, *L. principis-rupprechtii* forest, *L. chinensis* forest, *L. potaninii* forest, *L. sibirica* forest
	Pinus armandii forest	
	Pinus koraiensis forest	*P. koraiensis*, *P. koraiensis* and *Larix sibirica* mixed forest
	Pinus massoniana and *P. yunnanensis* forest	*P. massoniana* forest, *P. yunnanensis* forest, and *P. yunnanensis* Franch. var. *tenuifolia* forest
	Pinus sylyestris var. *mongolica* forest	
	Pinus tabuliformis forest	
	Tsuga, *Cryptomeria*, and *Keteleeria* forest	*T. dumosa* forest, *T. formosana* or *Picea morrisonicola* forest
	Other pines and conifer forests	*Juniperus rigida* forest, *Pinus densata* forest, *P. fenzeliana* forest, *P. griffithii* forest, *P. palustris* forest, *P. pumila* forest, *P. thunbergii* forest, *P. bungeana* forest, *P. densiflora* forest, *P. henryi* forest, *P. kesiya* forest, and *P. morrisonicola* forest
Broadleaf and conifer mixed forests	Mixed conifer and deciduous forest	*Chamaecyparis formosensis*, *C. obtusa*, *Fagus longipetiolata* and *Trochodendron aralioides* mixed forest, *P. koraiensis* and deciduous broadleaf mixed forest, *Tsuga chinensis*-broadleaf mixed forest, and *Tsuga dumosa*-broadleaf mixed forest
Deciduous broadleaf forests	*Betula* forest	*B. platyphylla* forest, *B. albosinensis* var. *septen-trionalis* forest, *B. albosinensis* forest, *B. ermanii* elfin forest, *B. luminifera* and *Populus adenopoda* forest, *B. utilis* forest
	Deciduous oak forest	*Quercus mongolica* forest, *Q. wutaishanica* forest, *Q. aliena* forest, *Q. dentata* forest, *Q. acutissima* forest, *Q. aliena* var. *acuteserrata* forest, *Q. variabilis* forest, *Q. baronii* forest, *Q. fabri* and *Q. serrata* Thunb. var. *brevipetiolata* mixed forest, *Q. serrata* forest, *Q. variabilis* and *Q. acutissima* mixed forest
	Nonmerchantable forest	*Tilia* and *Aceraceae* mixed forest

	Populus forest	*P. simonii* forest, *P. nigra* forest, *Populus, Salix* and *Ulmus* mixed forest, *P. davidiana* forest, *P. tremula* forest, *P. przewalskii* forest, *Chosenia arbutifolia* and *P. suaveolens* forest, *P. euphratica* open forest, *P. pruinosa* open forest, *P. davidiana* and *B. platyphylla* forest
	Other broadleaf forests	*Ulmus davidiana*, *Fraxinus mandschurica* and *Juglans mandshurica* mixed forest, *Robinia pseudoacacia* forest, *Malus sieversii* forest, *Salix matsudana* forest, *U. pumila* open forest, *Toxicodendron* spp. and *Acer mono* mixed forest, *Pteroceltis tatarinowii* forest, *Castanea seguinii*, *Quercus serrata* Thunb. var. *brevipetiolata* and *Platycarya strobilacea* mixed forest, *Liquidambar formosana* forest, *Platycarya strobilacea* and *Dalbergia hupeana* forest, *Alnus nepalensis* forest, *Q. variabilis* and evergreen broadleaf mixed forest, *Q. acutissima* and *Q. engleriana* mixed forest, *Q. acutissima*, *Q. variabilis*, *Phoebe zhennan* and *Cyclobalanopsis glauca* mixed forest, *C. multinervis* and *Fagus longipetiolata* mixed forest, *Lithocarpus cleistocarpus*, *Davidia involucrata* and *Tetracentron sinense* mixed forest, *C. glauca* and deciduous broadleaf mixed forest, *C. glaucoides* and *Platycarya longipes* mixed forest
Evergreen broadleaf forests	Other broadleaf forests	*Altingia chinensis* and *Rhodoleia championii* mixed forest, *Castanopsis delavayi*, *Cyclobalanopsis delavayi* and *Cyclobalanopsis glaucoides* mixed forest, *Castanopsis eyrei and C. carlesii* mixed forest, *Castanopsis faberi* and *C. calathiformis* mixed forest, *C. fargesii* forest, *C. fleuryi and Lithocarpus truncatus* mixed forest, *C. indica*, *C. platyacantha* and *Schima wallichii* mixed forest, *C. kawakamii* and *C. fleuryi* mixed forest, *C. orthacantha* forest, *C. platyacantha* and *C. tonkinensis* mixed forest, *C. platyacantha* forest, *C. sclerophylla* and *Cyclobalanopsis glauca* mixed forest, *Cryptocarya* spp. and *Castanopsis* spp. mixed forest, *Cyclobalanopsis glauca* and *Trochodendron aralioides* mixed forest, *Cyclobalanopsis oxyodon* and *C. gracilis* mixed forest, *Fagus longipetiolata*, *Lithocarpus glaber* and *Trochodendron aralioides* mixed forest, *Ficus* spp. and *Machilus kusanoi* mixed forest, *Lithocarpus cleistocarpus* forest, *Lithocarpus megalophyllus*, *Castanopsis carlesii* and *Beilschmiedia intermedia* mixed forest, *Lithocarpus variolosus* and *Schima argentea* mixed forest, *Lithocarpus xylocarpus*, *L. hancei*, *Cyclobalanopsis lamellosa* and *L. megalophyllus* mixed forest, *Machilus thunbergii* forest, *Quercus aquifolioides* forest, *Q. cocciferoides* forest, *Q. guyavaefolia* and *Q. longispica* mixed forest, *Q. pannosa* forest, *Q. pseudosemecarpifolia* and *Q. senescens* mixed forest, *Q. semicarpifolia* forest, and *Rhodoleia championii* and *Manglietia fordiana* mixed forest
	Tropical forest	*Bombax malabaricum* and *Albizia chinensis* mixed forest, *Dacrydium pierrei*, *Lithocarpus thalassica* and *Madhuca hainanensis* mixed forest, *Dipterocarpus retusus* and *Crypteronia paniculata* mixed forest, *Dipterocarpus turbinatus*, *Pandanus tectorius* and *Dysoxylum binectariferum* mixed forest, *Dysoxylum excelsum*, *Terminalia myriocarpa* and *Altingia excelsa* mixed forest, *Eberhardtia tonkinensis*, *Madhuca pasquieri* and *Altingia chinensis* mixed forest, *Excentrodendron hsienmu*, *Garcinia paucinervis* and *Cephalomappa sinensis* mixed forest, *Ficus altissima* and *Chukrasia tabularis* mixed forest, *F. benjamina*, *Barringtonia asiatica* and *Heritiera littoralis* mixed forest, *F. benjamina*, *Bischofia polycarpa* and *Dysoxylum excelsum* mixed forest, *F. microcarpa*, *Sterculia lanceolata* and *Schefflera octophylla* mixed forest, *Lysidice rhodostegia* and *Amesiodendron tienlinense* mixed forest, *Madhuca pasquieri* and *Altingia yunnanensis* mixed forest, *Parashorea chinensis* forest, *Podocarpus imbricatus*, *Hopea hainanensis* and *Adinandra hainanensis* mixed forest, *Schima wallichii* and *Liquidambar formosana* mixed forest, *Schoepfia jasminodora* forest, *Semecarpus reticulata* and *Phoebe nanmu* mixed forest, *T. hainanensis* and *Lannea coromandelica* mixed forest, *T. myriocarpa* and *Pometia pinnata* mixed forest

Supplementary References

1. Editorial Board of Vegetation Map of China. Vegetation Atlas of China. Science Press, Beijing (2001) [in Chinese].
2. Wang, J. S. *et al.* Biomass structure and allometry of *Abies nephrolepis* (Maxim) in Northeast China. *Silva Fenn.* **45**, 211–226 (2011).
3. Yang, J. M. & Fang, W. Y. Theoretical model for biomass of main tree species in Xiaoxing'an mountains. *J. Northwest For. Univ.* **39**, 46–48 (2011) [In Chinese with English abstract].
4. Wang, C. Biomass allometric equations for 10 co-occurring tree species in Chinese temperate forests. *For. Ecol. Manage.* **222**, 9–16 (2006).
5. Li, H. K. & Lei, L. C. Estimation and evaluation of forest biomass carbon storage in China. China Forestry Publishing Press, Beijing (2010) [In Chinese].
6. Han, S. Field observation and research database of ecosystems in China. Forest Ecosystem Volume, Changbaishan site (2001-2008). Science Press, Beijing (2012) [In Chinese].
7. Jiang, H. Plant ecology research. Insititute of Botany, The Chinese Academy of Sciences, Beijing (1992) [In Chinese].
8. Shen, Y. Z. *et al.* Study on the individual tree biomass of *Larix kaempferi* plantation in Xiaolong mountain, Gansu provinve. *For. Res*. **24**, 517–522 (2011) [In Chinese with English abstract].
9. Zhang, H. F. *et al.* A comparative study on biomass models for *Picea schrenkiana* in Xinjiang. *J. Northwest For. Univ.* **30**, 52–58 (2015) [In Chinese with English abstract].
10. Chen, T. R., Ma, Q. Y., Feng, Z. K. & Luo, X. Research on forest biomass in Xiaolong Mountains, Gansu Province. *J. Beijing For. Univ.* **29**, 31–36. (2007) [In Chinese with English abstract].

11. Fang, J. Y., Guo, Z. D., Piao, S. L. & Chen, A. P. Terrestrial vegetation carbon sinks in China, 1981–2000. *Sci. China Earth Sci.* **50**, 1341–1350 (2007).

12. Sang, W., Su, H. & Bai, F. Field observation and research database of ecosystems in China. Forest Ecosystem Volume, Beijing site (2000-2006). China Agriculture Press. (2010) [In Chinese].

13. Chen, L. Z. *et al.* Studies on Chinese arborvitae (*Platycladus orientalis*) forest and its biomass in Beijing. *Acta Phytoecol. Sin.* **10**, 17–24. (1986) [In Chinese with English abstract].

14. Lin, D., Lai, J., Muller-Landau, H. C., Mi, X. & Ma K. Topographic variation in aboveground biomass in a subtropical evergreen broad-leaved forest in China. *PLoS ONE* **7**, e48244 (2012).

15. Yang, H. *et al.* A study on the biomass and growth of three forestation models in Jishui region. *Acta Agr. Univ. Jiangxiensis* **26**, 164–168 (2004) [In Chinese with English abstract].

16. Wu, S. R., Yang, H. Q., Hong, R., Zhu, W. & Chen, X. Q. Studies on the biomass of *Pinus massoniana* plantations and its structure. *J. Fujian For. Sci. Technol.* **26**, 18–21 (1999) [In Chinese with English abstract].

17. Yao, Y. J., Kang, W. X. & Tian, D. L. Study of the biomass and productivity of *Cinnamomum camphora* plantation. *J. Cent. South For. Univ.* **23**, 1–5 (2003) [In Chinese with English abstract].

18. Wen, G. Y. Establishment and application of stand biomass scale of *Cunninghamia*. C*ent. South For. Inventory Plan.* **1**, 42–45 (1993) [In Chinese].

19. Fu, M. Y., He, J. Z., Song, L. H., Guo, H. H. & Yang, H. Z. The dynamic tree layer biomass of forest vegetation in the mountainous areas of Sanmenxia. *Henan Sci.* **34**, 332–337 (2016) [In Chinese with English abstract].

20. Feng, Z. W. *et al.* Determination of biomass of Pinus massoniana stand in Huitong county, Hunan province. *Sci. Silvae Sin.* **18**, 127–134 (1982) [In Chinese with English abstract].

21. Zhao, T. S. Study on material cycling in agroforestry complex ecosystem. I. Study on biomass of poplar in farm- and wood- land. *Rural Ecol. Environ.* **2**, 1–5 (1989) [In Chinese].

22. Wang, X. Y., Hu, D. & He, J. S. Biomass research of *Fagus engleriana* and *Quercus aliena* var. *acuteserrata* forest in Shennongjia forest district. *J. Cap. Normal Univ.* **28**, 62–67 (2007) [In Chinese with English abstract].

23. Li, Y. D. Comparative analysis for biomass measurement of tropical mountain rain forest in Hainan island, China. *Acta Ecol. Sin.* **13**, 314–320 (1993).

24. Luo, T. X. *et al.* Distribution patterns of aboveground biomass in Tibetan alpine vegetation transects. *Acta Phytoecol. Sin.* **26**, 668–676 (2002) [In Chinese with English abstract].

25. Chen, C. G. Biomass regression equations for broadleaf korean pine mixed forests. Yanbian For. Sci. Technol. **1**, 2–19 (1983) [In Chinese with with English abstract].

26. Shi, P. L., Yang, X. & Zhong, Z. C. Dynamics of population biomass and its density-dependent regulation in alder and cypress mixed forest. *Chinese J. Appl. Ecol.* **8**, 341–346 (1997) [In Chinese with English abstract].

27. Feng, Z. W., Wang, X. K. & Wu, G. Biomass and net productivity of the Chinese forest ecosystem. Science Press, Beijing (1999) [In Chinese].

28. Su, Y. M. A study on biomass and production of *Larix kaempfer* plantation. *J. Sichuan For. Sci. Technol.* **16**, 36–42 (1995) [In Chinese].

29. Jiang, H. Biomass and primary production in natural *Picea purpurea* forests. *Acta Phytoecol. Sin.* **10**, 146–152 (1986) [In Chinese with English abstract].

30. Zhang, Z. J. *et al.* Study on the biomass structure and distribution of natural secondary forest of *Pinus massoniana*. *J. Agr. Univ. Hebei* **29**, 37–43 (2006) [In Chinese with English abstract].
